# Adolescent neck pain: association with the use of mobile telephone

**DOI:** 10.1080/07853890.2021.1896436

**Published:** 2021-09-28

**Authors:** Beatriz Minghelli

**Affiliations:** a School of Health Jean Piaget Algarve, Piaget Institute, Silves, Portugal; b Research in Education and Community Intervention (RECI), Portugal

## Abstract

**Introduction:**

Neck pain has become a frequent problem in adolescents [1]. Multiple factors can be related to neck pain. The use of electronic devices has been suggested to increase the risk of neck pain, probably due to the static posture and with insufficient recovery after local muscle fatigue [2–5]. However, the data from a few studies are contradictory [1,6]. Thus, this study aimed to evaluate the prevalence of neck pain in adolescents and their association with mobile phone use.

**Materials and methods:**

This is a cross-sectional study approved by Research in Education and Community Intervention (RECI) research centre. Written informed consent were obtained from all parents or guardians of the students participating in the study. The sample was comprised of 206 students, being 109 (52.9%) boys and 97 (47.1%) girls, aged between 12 and 19 years old (14.67 ± 1.51), enrolled in the 8th (*n* = 104; 50.5%) and 10th (*n* = 102; 49.5%) grades of the D. Martinho Castelo Branco, elementary school, and Poeta António Aleixo, high school, both in Portimão, south of Portugal. The measurement instruments included a self-report questionnaire of neck pain, including questions about the time spent using mobile phones, and a practical test to evaluate the posture with the use of the mobile phone during the sending of a text message (a standard posture was established).

**Results:**

One hundred and fourteen (55.3%) adolescents reported neck pain at some point in their life (lifetime prevalence), 16 (7.8%) referred neck pain at the moment of evaluation and 75 (36.4%) in a 6-month period. Regarding the use time of the mobile phone per week, 8 (3.9%) students reported that they do not have a mobile phone, 18 (8.7%) use until 5 h, 32 (15.5%) between 6 and 10 h, 44 (21.4%) between 11 and 15 h, and the majority of the adolescents (*n* = 104; 50.5%) reported that they use the mobile phone for a period equal to or greater than 16 h per week. One hundred ninety-seven (95.6%) students used the mobile phone incorrectly with flexion of the cervical spine. The adolescents who used the mobile phone more than 10 h per week showed a 1.58 times greater risk of neck pain (95% CI: 0.59–4.23; *p* = .360) than those who use equal or less than 10 h, and adolescents who use the phone in a wrong way had a 1.15 times higher risk of neck pain (95% CI: 0.28–4.75; *p* = .845) than those who used it correctly.

**Discussion and Conclusions:**

The data obtained in this study showed a high rate of neck pain in the analysed sample of adolescents. Myrtveit et al. [6] used data from the population-based study and their results showed that 1,79 (20%) of the total 8,990 adolescents reported neck pain during the last 6 months and the Shan et al. [5] showed a prevalence of 40.8% in 3016 students, in the same period. Regarding mobile phone use as a risk factor, it was observed that adolescents who use the mobile phone for many hours and those who use it with the wrong posture showed a greater probability of neck pain, however, no significant associations were observed. It is necessary to carry out more studies to expand the knowledge of neck pain associated with the time and use of screen-based activities and on their prevention.
